# SACT: A New Model of Covert Communication Based on SDN

**DOI:** 10.3390/s20247300

**Published:** 2020-12-19

**Authors:** Leyi Shi, Yuxiao Song, Zhiyu Xue, Yihao Liu, Honglong Chen

**Affiliations:** 1 College of Computer Science and Technology, China University of Petroleum, Qingdao 266580, China; z18070016@s.upc.edu.cn (Y.S.); s15070756@s.upc.edu.cn (Z.X.); s18070009@s.upc.edu.cn (Y.L.); 2 Guangxi Key Laboratory of Cryptography and Information Security, Guilin University of Electronic Technology, Guilin 541004, China; 3 College of Control Science and Engineering, China University of Petroleum, Qingdao 266580, China; chenhl@upc.edu.cn

**Keywords:** watermarking, SDN, covert communication, dynamic protection, security analysis

## Abstract

Anonymous tracking technology of network watermarking is limited by the deployment of tracking devices in traditional network structure, resulting in poor scalability and reusability. Software Defined Network (SDN) boasts more freedom thanks to its separation of the control plane from the data plane. In this paper, a new anonymous communication tracking model SDN-based Anonymous Communication Tracking (SACT) is proposed, which introduces network watermarking into SDN and combines IP time hidden channel and symbol expansion technology. In addition, we introduce a hopping protection mechanism to improve the anti detection ability of the watermark as well. The experimental results show that in a variety of simulated network environments, SACT achieves excellent detection rate and bit error rate, thus it is sufficient to determine the communication relationship between the two parties. Meanwhile, SACT solves the deployment problem of anonymous tracking and improves the availability and scalability of covert communication.

## 1. Introduction

Anonymous communication system is a privacy protection technology that hides the communication content or both parties of communication by data forwarding, content encryption, traffic confusion and other measures. The purpose of anonymous communication technology is hiding information, thus attackers, censors and firewalls may be the enemies of anonymous communication. Most of the anonymous communication usually use a multi hop reverse proxy or resource-shared storage to mask the real address and ensure that the anonymous services are not traceable. Researchers have designed a variety of anonymous communication systems such as Reiter et al. [1], Dingledine et al. [2] and Cranor [3].

Most of these systems use reroute technology for covert communication. These systems insert one or more intermediate nodes in the communication path between the sender and the receiver and rewrite the IP address and other related information to hide the source, destination and entry-exit relationship of the packet while forwarding the data packet. In this way, the IP address obtained by the attacker is not real so that the real communication relationship cannot be inferred. Because of the difficulty in discovering communication nodes and determining communication relationships, hidden channels are gradually adopted by malware to establish anti forensics mechanism or abuse communication services.

By marking and comparing the communication packets of the users, the tracking technology of communication layer analyzes the sending and receiving packets of the communication parties and confirms the correlation between them. These tracking technologies have great advantages in general resource requirements and application scope. A hidden communication path in the communication layer can be created by adding private data to the different characteristics of network traffic. Unused fields in the packet header, packet error rate, and jitter all can be carriers. As an active traffic analysis method [4] in the communication layer, network watermarking [5] can be used to trace the malicious users who communicate illegally through anonymous channels and network attack source through the gangplank chain.

However, in the traditional network structure, if the configuration of network equipment needs to be modified after deployment, it will be a complex, costly, and time-consuming work. Due to the complexity of the algorithm, the above method cannot be directly deployed in the traditional network, additional devices such as watermark embedder need to be added in the network environment. It is quite difficult to modify the network equipment which has already been deployed. More importantly, traditional watermarking technology needs to embed deployment points in the network, which is uncomplicated to arouse the vigilance of criminals.

The emergence of Software Defined Networks (SDN) [6] promotes the development of anonymous communication tracking technology. SDN separates the control plane from the data plane and improves the programmability of the network. The data layer and control layer use open unified interface (such as openflow [7] protocol) for interaction. The tracking scheme in traditional network can be directly deployed in the data layer of SDN, which can effectively reduce the equipment load. SDN not only solves the problem of watermark deployment but also eliminates the side effects of embedding watermark through coding. For attackers, it is hard to be suspicious and vigilant to the watermarked data stream, which is of great significance to the anonymous tracking system.

In this paper, we concentrate on the combination of network watermarking technology and SDN to improve the concealment and security of SDN data transmission. There are three challenges:

- Security: Before the data stream enters the network, the watermark is embedded into the network data flow through the SDN controller. In order to avoid being detected by attackers and reduce data corruption, a secure and rigorous watermark coding scheme is needed.
- Integrity: In the network, the communication environment is not invariable. Considering that the packet embedded with watermark is affected by network jitter during transmission, its interval time may be offset when it arrives at the destination. Therefore, it is necessary for us to find a solution to guarantee the integrity of data transmission.
- Concealment: Embedding watermarking information into network data stream will affect original delay sequence of packets, which is obviously different from normal data flow. It is necessary to improve the concealment of watermark in the transmission process.

In order to solve these problems brought about by the introduction of watermarking in SDN, a new SDN model, SDN-based Anonymous Communication Tracking (SACT), which uses IP hidden channel [8] to write watermark to SDN, is proposed in this paper. Combining convolutional coding method and *L-Bits to N-Packets* model, the encoding embedding and extracting decoding strategy of network watermarking were designed, so as to improve self correction ability and channel capacity. In addition, anti-interference processing mechanism is added to the system to reduce the influence of network jitter on the delay sequence of watermarking. Finally, the anti detection strategy of watermark is put forward to hide the watermark features with the aid of the distributed simulation mechanism. Introducing a dynamic protection mechanism [9] to dynamically change the mapping, reduces the possibility of being detected by attackers and improves the anti detection ability of the watermark, thus realizing the covert tracking of anonymous communication.

The main contributions of this paper are summarized as follows:

- We designed a method to embed watermark by encoding and decoding in SDN and established a model of covert communication based on SDN by integrating watermark technology into SDN.
- The delay problem resulted by network jitter in the transmission of watermark was solved, improving the anti detection ability of watermark in the transmission.
- We designed SACT, an tracing prototype system about anonymous communication based on SDN. We analyzed and tested the accuracy rate, false alarm rate and anti detection ability of watermark tracking information, and the results of the experiment prove the effectiveness of our system.

The rest of this paper is organized as follows: In Section 2, we review the existing solutions for SDN and anonymous communication. Section 3 describes the security analysis process of our SACT scheme. The system model is defined in Section 4. The experiments are conducted in Section 5. In Section 6, we describe the shortcomings of our experiment and the future work. Furthermore, Section 7 makes a conclusion for this paper.

## 2. Related Work

In this section, we conduct a literature review on anonymous communication.

Most of the early traffic layer tracing techniques are passive. These techniques monitor the data of both sides of the communication, and then calculate the similarity according to the selection algorithm. The communication relationship between the two parties was judged by the confidence interval threshold [10,11].

In order to solve the shortcomings of passive tracking techniques, researchers have proposed active traffic analysis and tracking technology, which is represented by network flow chart. The tracking is carried out by hidden channel information such as the time interval between packets and the rate of the target traffic. Raghunathan et al. [12] have disclosed the system and method for anonymizing data. This method includes intercepting communication, identifying sensitive data in communication, sending data to anonymous engine for shielding, and receiving masked data from anonymization engine and sending it to client devices. He provided a systematic theoretical structure and method for anonymous communication. Chase et al. [13] designed a new method of end-to-end anonymous messaging, which greatly reduced the time cost.

Emura et al. [14] proposed a secure and anonymous communication protocol to solve the problem of authentication mechanism of anonymous communication channel. The protocol hides the user information by proxy entity which communicates in the protocol. Concept verification is introduced to demonstrate the feasibility of the protocol and analyze its performance. The secure anonymous communication between users and service provider are realized. Yang et al. [15] proposed a new anonymous network authentication protocol, which used pseudo identity mechanism and identity based elliptic curve system to achieve anonymity, robustness, and efficiency.

Kang [16] researched anonymous communication in the network topology. He proposed many improved node selection and entry protection algorithms under the theoretical framework. However, the practicability of these algorithms in real-time anonymous systems was not clear. Castillo et al. [17] researched two kinds of covert timing channel (CTC) and provided a method of sending information in secret by modulating time. Covert communication was carried out by hiding message content and providing confidentiality in this method. The detection capability, robustness and communication speed of the system were tested experimentally, which provided a new way to realize anonymous communication. Nia et al. [18] systematically discussed the current situation and future research work of anonymous communication system, and introduced the methods of anonymous network modeling, anonymization, and building communication channels. A covert communication system was proposed by Xu et al. [19], in which a nonzero mean Gaussian sequence was used as a random carrier and its mean was modulated by a covert binary bit.

Zhang et al. [20] proposed a watermarking technology based on packet order rearrangement (PROFW), which combined error correction coding technology. The watermark information is embedded in the order of packet sending in the data stream, and the watermark detection and concealment are improved. Then, a spread spectrum watermarking scheme based on time interval was proposed [21]. Through the spread spectrum idea in digital communication, pseudo noise (PN) code was used to complete the expansion coding of watermark information and realize the random arrangement of interval sequence. The disadvantage is that the robustness of watermark is reduced. Then, a non blind stream watermarking technology was implemented based on the spread spectrum principle of digital communication, which had high concealment and can effectively resist time analysis attack [22]. However, the coding mapping scheme needs to be negotiated in advance in this method.

He et al. [23] proposed the theoretical basis of hiding tracking information into time information, which had become the research focus of network watermarking technology. Zhang et al. [24] proposed a watermarking technology based on slot centroid flow. The watermark information was embedded by using the slot centroid of the data stream. This method used fixed values to modify the delay sequence, which was not strong in concealment and had weak resistance to time disruption. Therefore, it cannot handle time analysis attack, multi stream attack, and mean square autocorrelation attack. Lu et al. [25] introduced some different schemes of watermarking in anonymity systems and discussed some attacks against it, putting forward some ideas for improving watermarking.

Zear et al. [26] proposed a secure multiple watermarking technique based on discrete wavelet transforms (DWT), discrete cosine transform (DCT), and singular value decomposition (SVD). This method resisted different signal processing attacks and performed well in robustness, imperceptibility, capacity, and security. In order to improve the security of the watermark, Thakur et al. [27] applied the encryption algorithm based on chaos to the watermark image. The algorithm had exceptional robustness and enough security to deal with all kinds of attacks.

In order to further improve the robustness of watermarking scheme, Geng et al. [28] proposed a removal attack of convolutional neural network (CNN). Based on 2D frictional fryer transformation technique (FRFT), Shahabadkar et al. [29] proposed a new watermarking technology, which can survive various image processing attacks and protect the embedded digital content. The system introduced an attack model designed by two common image processing attacks to evaluate the strength of the proposed watermarking scheme.

The technology of increase and decrease with pseudo random of packet interval delay [30] had a good anti detection ability, but it performed poorly in the network environment with poor stability, resulting in poor accuracy. The sliding window algorithm was applied to the watermarking technology, which improved the resistance and concealment of the carrier data stream to network jitter [31]. However, accuracy and fault tolerance still need to improve.

Benabbou et al. [32] analyzed the positive and negative impacts of SDN on network security, described the availability access control and application-oriented security status of SDN, and emphasized the opportunities and challenges brought by SDN to network security. Marin et al. [33] put forward two new attacks in SDN and expounded the countermeasures against system vulnerabilities of SDN.

The optimization of watermarking algorithm is of great significance to the development of anonymous communication. However, these methods only improve the security of the watermark but ignore the performance limitation caused by the difficulty of deployment. In this paper, aiming at the deployment of traditional anonymous tracking model, the watermarking technology is integrated into SDN to improve the concealment of data transmission and solve the deployment problem of anonymous tracing.

Our contribution differs significantly from the above work:

- The convolutional code is applied to watermark tracking in our system, which can effectively reduce error code and improve robustness. In addition, the encoded watermark is compressed and converted to improve channel utilization. It not only improves the security of watermark but also ensures efficiency. Most of the other schemes only focus on the security of watermark but ignore the efficiency of watermark transmission channel.
- The combination of watermark and SDN effectively solves the deployment of watermark tracking scheme, reduces the cost of deployment.
- Our system dynamically transforms the coding mapping scheme, effectively prevents the interception and analysis of the coding scheme.

It is of great significance to solve the problems of attack source tracking, network monitoring, and forensics.

## 3. Materials and Methods

In this section, the overall strategy of the system is analyzed, including the research of watermark conversion strategy and the analysis of anti detection strategy. Furthermore, the tracking watermark is analyzed and optimized. We improve the anti detection ability of the tracking watermark through the distributed simulation mechanism and analyze the effectiveness of system. An anonymous communication tracing strategy based on SDN is proposed.

### 3.1. Watermark Conversion Analysis

#### 3.1.1. Watermark Modulation Analysis

Network watermarking is divided into non blind watermarking and blind watermarking [34]. The network watermark will be affected by many factors such as network fluctuation when it is transmitting. The robustness of network watermarking is the primary factor to be considered, and the encoding method of watermark information is the key to the robustness.

According to Shannon theorem, we can reduce the error probability exponentially by increasing the code length when the channel capacity and transmission rate are fixed [35]. With the increase of code length, the complexity of encoding and decoding increases, as well as the time of encoding and decoding.

Convolutional code is an error control channel coding with superior performance. The data encoding process is related to binary polynomial sliding [36]. Convolutional code encodes *m-bit* information sequence into *n-bit* information. The encoded *n-bit* information is not only related to the *m-bit* source information of this group but also to the bit information of the first *L-1* group at other times. The length of *L* group code element information associated with the encoding process is called the constraint length of convolutional code. Convolutional code is usually represented by triples *(n, m, L)*, where *n* is the output bit information, *m* is the input bit information, *L* is the length or memory depth of the constraint code group, and the ratio of *m* to *n* is called the code rate of convolutional codes.

**Figure 1:**
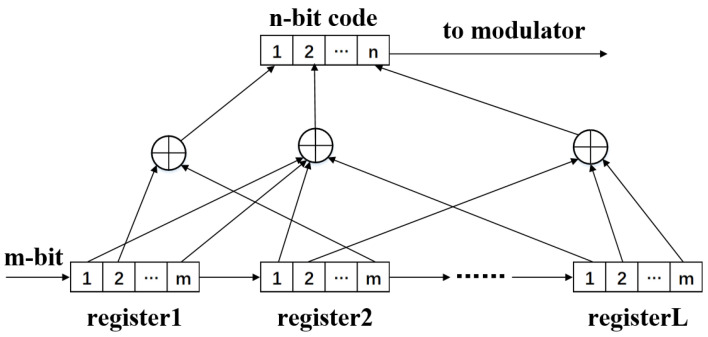
Structure of the convolutional code encoder.

The encoder structure of convolutional code is shown in Figure 1, *L* registers indicate that there are *L-level* bits information. Each register can input *m* bits information, then there are m * L bits information in total, and *n* bits information is output to modulator. The output information is not only related to the *m* input information of the current packet but also to the bit information of the previous *L-1* group. The error correction ability of convolutional codes increases with the increase of *L*, and the error rate decreases exponentially with the increase of *n* [36].

**Figure 2:**
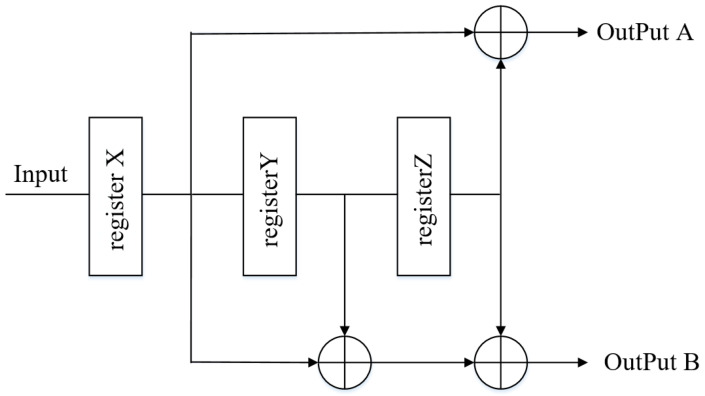
Method of the convolutional code encoder.

The original binary watermark information enters the encoder register through the input end in turn. Each encoding register stores a block of input data with an initial value of 0. As shown in Figure 2, there are three *Modulo-2* Adders (equivalent to an Exclusive OR(XOR) gate) in the encoder, and the operation mode is *0 + 0 = 0, 0 + 1 = 1, 1 + 0 = 1, 1 + 1 = 0*. The addition operation is performed on *3-bit* raw data. Then, the data in the register is sent to the next register (x→y,y→z) and continue to the output to obtain the data to be transferred.

According to Equation (1), the encoded convolutional code data is output at the output end.

(1) $$\begin{array}{c} A=X+Z\\ B=X+Y+Z \end{array}$$

The original watermark information is input to the encoder in sequence and the data stored in the three registers is the input values at different time points. Register *X* stores the current block symbol data, register *Y* stores the data of the previous cycle, and register *Z* stores the data of the previous cycle. Each group of information data of convolution expanded watermark are related to the past information data, retain memory effect, and improve the robustness of watermark information. The coding complexity can be expressed by the length of the coding constraint: n∗L, the time complexity is $O(n^{2})$ [36].

After the convolutional code is applied to the network watermark coding, the anti-interference ability of the network watermark is improved. However, the length of the watermark information is multiplied by the convolutional code. The *L-Bits to N-Packets* model is introduced [37] to solve the problem of channel capacity reduction caused by the introduction of convolutional code.

The arrival delay between *n* packets is used to represent the *L-bit* string in encoder. Only the *L-bit* string in the code group is affected when an error occurs during decoding. Suppose that the sender sends two packets in the order of *a* and *b*, and the sending time is $t_{a}$ and $t_{b}$, then the time interval between the two packets is $T=t_{b}-t_{a}$. Each *L-bit* watermark string is mapped to *N* packets interval arrival delay $\left(T_{1},T_{2},\cdots ,T_{3}\right)$ in the *L-Bits to N-Packets* coding. The value of $T_{i}$ comes from the set $E=\left(T:T_{min}+n\cdot \Delta ,n=0,1,\cdots \right)$, $T_{min}$ represents the minimum interval delay in the interval delay of packet, and Δ represents the step of arrival delay between adjacent packet, e.g., in the *2-Bits to 1-Packets* coding scheme, taking $T_{min}=60$ ms and Δ=20 ms, the binary string ‘10’ is mapped to the interval arrival delay $T_{1}$ = 60 ms. The binary strings 01, 11, and 00 are mapped to $T_{1}=80$ ms, 100 ms, and 120 ms respectively.

The results are shown in Table 1. The length of the code table is appropriate, thus the encoder is simple to implement—it does not take up too many resources. Therefore, the encoding length is reduced and the channel capacity is increased.

#### 3.1.2. Watermark Demodulation Analysis

There are constraints between groups of convolutional codes. Viterbi decoding reduces decoding error rate [38] by using the algebraic structure of convolutional code and the probabilistic statistical properties of the channel. When the inter group constraint of convolutional codes is small, the decoding speed of Viterbi decoding is fast and the algorithm complexity is relatively low. The algorithm complexity Viterbi decoding is $O(n*d^{2})$, *n* is the number of sequences, and *d* is the length of sequences [36].

After extracting the binary watermark sequence from the data stream, our system compares it with all sending sequences. Then finding a path with the smallest Hamming distance [39] between the corresponding coding sequence and the received sequence of watermark information. The symbol information corresponding to this path is taken as the encoding output, which is the watermark information sequence before convolution expansion.

In order to verify the correlation between the extracted watermark sequence *B* and the original watermark sequence *A*, we need to calculate the similarity of the two watermark sequences and set their threshold range. Then the similarity is compared with the threshold parameter of the confidence interval. If the similarity calculation result is within the confidence interval, it is determined that the watermark information embedded in the current detected data stream is associated with the source data stream. That is, there is a communication relationship between the current detected communication parties, otherwise, there is no communication relationship.

### 3.2. Watermark Anti Detection Analysis

#### 3.2.1. Distributed Simulation Strategy

After the tracking information is converted into the packet delay sequence according to the watermark encoding scheme, the delay of the information is affected, and the statistical law is different from that of the normal data flow. The interval arrival delay of packets in the network is a random variable in accordance with normal distribution [40]. Distributed simulation methods obtain the approximate reversible transformation function by detecting the statistical distribution of normal interval arrival delay in the network. The encoded packets are modulated according to this transformation function. In this way, the distribution pattern of packets is similar to the distribution of normal data flow in the network, thus avoiding detection by attackers.

Suppose that controller obtains the delay sequence $\left(T_{1},T_{2},\cdots ,T_{n}\right)$ after watermark information is encoded and modulated. It is transformed into a set of random variables $\left(tran\left(T_{1}\right),tran\left(T_{2}\right),\cdots ,tran\left(T_{n}\right)\right)$ by invertible transformation function, and it accords with the distribution function of normal interval sequence in the network. tran(x) is a reversible transformation function. After receiving the carrier data stream, the receiver can restore the $\left(tran\left(T_{1}\right),tran\left(T_{2}\right),\cdots ,tran\left(T_{n}\right)\right)$ sequence to the $\left(T_{1},T_{2},\cdots ,T_{n}\right)$ sequence through $tran^{-1}\left(x\right)$ function to confuse the attacker.

According to the law of probability integral transformation in probability theory:

1. If the distribution function F(x) of *X* is continuous, then Y=F(x) is uniformly distributed on the interval (0,1).
2. If *Y* is uniformly distributed in the interval (0,1), let $X=F^{-1}\left(Y\right)$, then *X* is a random variable whose distribution function is F(x) for any distribution function F(x).

Assume that the current interval sequence of network data flow accords with normal distribution function F(x), and $T_{i}\left(i=1,2,\cdots ,n\right)$ is within U(0,1). Then the transformation function tran(x) and its inverse function $tran^{-1}\left(x\right)$ are defined as:

(2) $$\begin{array}{c} tran\left(x\right)=F^{-1}\left(x\right)\\ tran^{-1}\left(x\right)=F\left(x\right) \end{array}$$

The arrival delay sequence $\left(T_{1},T_{2},\cdots ,T_{n}\right)$ of the original interval is transformed into a delay sequence that follows the current network delay distribution function F(x) through the transformation function tran(x). The solution of the transformation function can be changed into the solution of the inverse function $X=F^{-1}\left(Y\right)$ of the delay distribution function F(x) in current network. In this paper, the approximate reversible transformation function of the inverse function $F^{-1}\left(x\right)$ of the network delay distribution function is constructed by using the discrete approximation method in the stochastic simulation method [41]. Then, the delay sequence is modulated to accord with the delay distribution law of network transmission.

#### 3.2.2. Mapping Synchronization Strategy

The mapping relationship between watermark information and binary code (i.e., binary code table) is stored in watermark embedder and extractor. In this paper, the End Hopping [42] is used as a reference. The coding mapping relationships change dynamically according to the time, so that the distribution of watermark information is more complex to confuse attackers. The continuous transformation of coding mapping relationship improves the diversity of watermark carrier data stream distribution. Correctness of time synchronization is the key to ensure the basic function of the model system.

At present, the popular synchronization methods include ACK *(Acknowledge Character)* response synchronization, timestamp synchronization, and strict clock synchronization [43]. Each of the three methods has characteristic advantages. The accuracy of ACK response synchronization depends on ACK acknowledgement packets, and a congested network may lead to inefficient ACK packet transmission. Timestamp synchronization is relatively secure and does not change due to the network jitter, but it is complex, expensive, and inefficient to implement. Strict clock synchronization is the simplest and most efficient method, but this method is susceptible to NTP *(Network Time Protocol)* server, which can lead to error synchronization.

In this paper, considering the efficiency of synchronization, the strict clock synchronization method based on NTP is used to realize the synchronization of the controller code mapping relationship. It can not only be used to evaluate the clock offset between multiple terminals but also to estimate the RTT *(Round-Trip Time)* of data packets. NTP synchronization is a mature and widely used synchronization method, and there are mature solutions to the clock skew problem.

Controller sends synchronization request to NTP server. The current encoding relationship is determined based on the timestamp returned by the NTP server. In this way, the diversity of encoding is realized and the anti detection ability of watermark is improved.

#### 3.2.3. Effectiveness Analysis

After the watermark information is transformed into a delay sequence and embedded to data stream, the delay distribution state of the data stream will change. As the number of embedded packets increases, the distribution may gradually tend to uniform distribution.

Suppose that *N* is the number of all packets sent during communication, and *n* is the number of packets that the attacker needs to capture to determine the current distribution state of flow delay.

In the absence of distributed simulation mechanism, watermark delay sequence does not accord with normal distribution. If attackers attack the network, the success rate is shown in Equation (3).

(3) $$\begin{array}{c} \rho =(N-n)/N \end{array}$$

In practical or uncontrolled environments, the delay distribution of the data stream embedded with watermark information is uncertain. Its distribution also tends to be uniform rather than normal in the case of sufficient data packets. So Equation (3) is effective in practical situations. Success rate ρ only depends on the number of packets the attacker needs to capture. The lower the number of packets needed, the higher the success rate. As the number of packets captured by the attacker increases, the watermark is more likely to be detected.

When the distributed simulation mechanism works, the distribution state of delay interval obtained by attackers is fitted with that of the normal data flow. The attacker cannot confirm the existence of the watermark.

When the attackers discover that the delay information of the current detection data stream is abnormal, it is assumed that there are m(m≥2) kinds of code mapping relations preset in the controller, and the dynamic change time slot of the mapping relationship is τ. The time taken by the attacker to obtain and analyze the mapping relationship is *t*, and the total communication time is *T*.

In the absence of a dynamic synchronization mechanism, the mapping scheme remains unchanged during communication. The success rate of the attacker to confirm the watermark information by acquiring and analyzing the mapping relationship is shown in Equation (4). It can be seen that the success rate is only affected by the time the attackers get the mapping relationship. Since the mapping relationship remains unchanged, the success rate of the next attack directly increases to 1 after the attacker succeeds.

(4) $$\begin{array}{c} \alpha =(T-t)/T \end{array}$$

Controller can make pseudo random selection from the preset coding mapping relationship when dynamic synchronization mechanism exists. The success rate of the attacker to obtain the mapping information is shown in Equation (5).

(5) $$\begin{array}{c} \beta =(\tau -t)/mT(\tau \geq t,m\geq 2) \end{array}$$

It is obvious that if β<α, the smaller the dynamic time slot is, the closer (τ−t) is to 0. When the dynamic change time slot is small enough, the attacker cannot restore the mapping relationship, so as to avoid the leakage of watermark information. If the dynamic time slot is large, the attacker can successfully resolve the code mapping relationship. The system can still reselect a new mapping relationship through the synchronization algorithm after the end of the current dynamic time slot, so as to avoid the attackers from attacking again.

Through the above analysis, the current anti detection strategy can effectively improve the concealment of watermark information and resist the detection of attackers.

## 4. System Model

**Figure 3:**
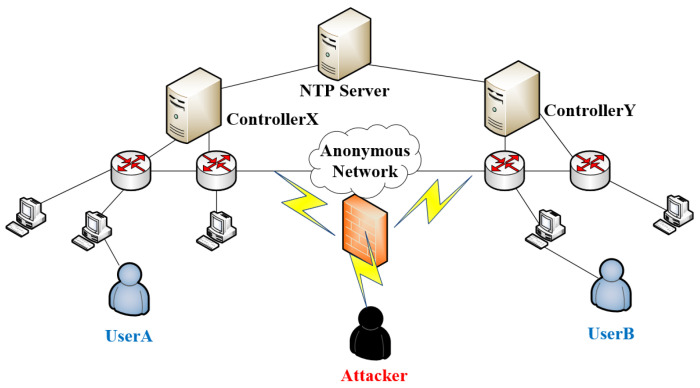
SDN-based Anonymous Communication Tracking (SACT) system model.

In this section, we propose an anonymous communication model SACT based on SDN, which is composed of NTP server, SDN controller, host, and SDN link. As shown in Figure 3, there are two participants: an illegal attacker and a legitimate user. By embedding watermark, the data transmission is invisible for the attacker.

Controller *X* embeds the watermark *W* into the packet sent by *A* and sent it to the communication network. After the packet arrives at the network switch where *B* is located, the flow table action is triggered to notify controller *Y* to extract the watermark $W^{\prime }$ from it. If the similarity between $W^{\prime }$ and *W* is less than the threshold η, then *A* and *B* are considered to have communication behavior.

**Figure 4:**
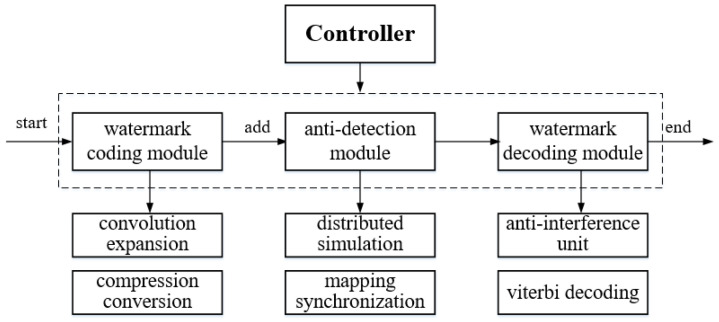
Controller module.

The controller modulates and demodulates the watermark information, including three general modules as shown in the Figure 4.

**Figure 5:**
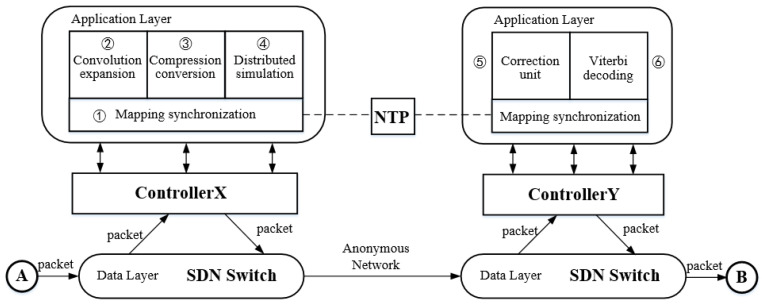
Application Components of SACT.

We describe the application components of our system in the Figure 5. Mapping synchronization, convolution expansion, compression conversion, and distributed simulation modules are deployed in Controller X, and mapping synchronization, correction unit, and compression conversion modules are deployed in Controller Y. The modules are as follows:

- Mapping synchronization: Communicate with the NTP server to get the current encoding mapping relationship. The module is deployed in both controller *X* and *Y*, and the corresponding mapping is required for both encoding and decoding.
- Convolution expansion: As the core component of the system, convolution expansion module carries out convolutional encoding of the watermark information. After completion, this module sends the watermark to the compression conversion module.
- Compression conversion: In order to solve the problem of channel capacity reduction caused by the introduction of convolutional code, this module compacted and converted the watermark information after convolutional coding to improve the efficiency.
- Distributed simulation: In order to make the arrival delay of the packet interval after adding the watermark close to the normal network, the module fitted the delay sequence corresponding to the watermark information after compression conversion.
- Correction unit: After controller *Y* receives the packet, its arrival delay is corrected to obtain the initial sequence.
- Viterbi decoding: Decode the watermark to judge the communication relationship between *A* and *B*.

### 4.1. Watermark Embedding Module

SACT system embeds watermark information by controlling the network data flow and capturing the required packet flow. TCP protocol is a reliable communication protocol for connection. The application layer protocols that it supports include: HTTP/HTTPS, FTP, Telnet, SMTP, POP3. UDP protocol is a connectionless and unreliable communication protocol, which can provide excellent instant information processing. The application layer protocols that it supports include: DHCP, NTP, DNS, NFS, SNMP. To reduce the load of SDN control, our system only writes watermark information through TCP/UDP protocol.

The workflow of the module is as follows:

1. Setting the sending of flow table in SDN switch. After receiving TCP/UDP protocol data packet, the switch uploads the key information of data packet and switch port and other information to the controller for further processing through *Packet-In* message. If the switch receives other protocol packets, it directly forwards it according to the flow table.
2. After receiving the message, the SDN controller converts the switch port and other information into the binary bit stream *B* according to the coding mapping relationship as the initial watermark information.
3. The binary bit stream *B* is extended by convolutional code to obtain sequence *E*, which improves its self error correcting ability. We use the *L-Bits to N-Packets* coding model to transform it into the interval arrival delay sequence *M*. The controller notifies the switch to send packets according to the delay sequence by *Packet-Out* message.

#### 4.1.1. Convolution Extension

**Figure 6:**
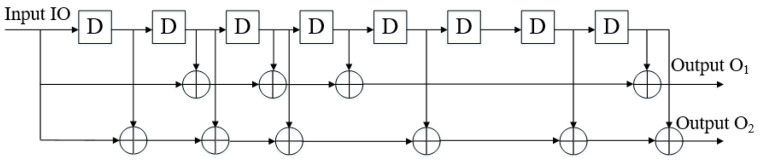
Convolutional code encoder.

To improve the robustness of watermark in network transmission, we use convolutional coding (2,1,9) with a bit rate of 1/2 and a constraint length of 9 to embed the binary watermark information into the data stream for extended output, as shown in Figure 6.

The watermark information sequence is input by *Input-I0* and output through $O_{1}$ and $O_{2}$ after coding in encoder. The corresponding sub generator of $O_{1}$ is $G_{1}=561$, and $O_{2}$ is $G_{2}=753$. There are eight registers in this encoder. If the input data stream size is *n* bits, the output data stream is (L=2n+16) bits after encoding.

The corresponding generators of $O_{1}$ and $O_{2}$ are $G_{1}$ and $G_{2}$ respectively, their corresponding binary matrices are as follows:

$$\begin{array}{c} G_{1}=101110001\\ G_{2}=111101011 \end{array}$$

We set the initial value of the encoder register to 0 and set the input watermark sequence. The output sequences of each output end after passing through the encoder are as follows:

$$\begin{array}{c} O_{1}=G_{1}*s=101110001*110110=100110111010110\\ O_{2}=G_{2}*s=111101011*110110=110011110010010 \end{array}$$

After combining the output sequences of $O_{1}$ and $O_{2}$, the resulting of extended sequence is as follows:

$$\begin{array}{c} \left(11,01,00,10,11,01,11,11,10,00,11,00,10,11,00\right) \end{array}$$

The convolution operation of vector can be realized by conv() function in MATLAB. Therefore, the implementation of convolution coding in MATLAB are as follows:

(6) $$\begin{array}{c} O_{1}=mod\left(conv\left(s,G_{1}\right),2\right)\\ O_{2}=mod\left(conv\left(s,G_{2}\right),2\right) \end{array}$$

Then the *m* file program is compiled into jar package to encode the watermark information sequence.

#### 4.1.2. Compression Conversion

In this paper, *L-Bits to N-Packets* coding scheme is introduced to solve the problem of channel capacity reduction. The selection of *L* and *N* size is related to the data transmission rate in this scheme. According to Sellke et al. [44], *8-Bits to 3-Packets* coding scheme is chosen to simplify the encoding and decoding algorithms and improve their efficiency.

In the *8-Bits to 3-Packets* coding scheme, every *8-Bits* binary code is mapped to a non-negative integer vector $\left(n_{1},n_{2},n_{3}\right)$, where $n_{1}+n_{2}+n_{3}\leq 10$. Then, vector $\left(n_{1},n_{2},n_{3}\right)$ is mapped to interval arrival delay sequence $\left(T_{1},T_{2},T_{3}\right)$ according to Equation (7).

(7) $$\begin{array}{c} T_{i}=T_{\min}+n_{i}\Delta ,i=1,2,3 \end{array}$$

For example, the mapping vector $\left(n_{1},n_{2},n_{3}\right)$ of the *F* is (1,1,5). If the minimum interval arrival delay of the current network is $T_{\min}=50\mathrm{ms}$ and the interval sequence step size is Δ = 10 ms, then it can be mapped into interval arrival delay sequence $\left(T_{1},T_{2},T_{3}\right)=\left(50+10,50+10,50+50\right)=\left(60,60,100\right)$. To embed them into the packet stream, four packets need to be sent. Their sending time is $\left(t_{0},t_{1},t_{2},t_{3}\right)=\left(0,60,120,220\right)$, and the total time of embedding 8−bit binary information is 220 ms.

A mapping table with the same size and content is maintained in the controller. The conversion between binary sequence and vector is carried out combined with Equation (7). By the same encoding table based on $T_{\min}$ and Δ, the SDN controller keeps the mapping relationship between the binary sequence and the delay sequence in the sender and receiver.

### 4.2. Anti Detection Module

To enhance the anti detection ability of watermarking, distributed simulation and mapping synchronization are proposed in SACT.

#### 4.2.1. Distributed Simulation

It is regular for the data flow of normal distribution in the network, and the distribution function is F(x). In SACT, the reversible transformation function tran(x) of distribution function F(x) of delay sequence is constructed by random simulation method. Then modulating the delay sequence corresponding to watermark information. The steps are as follows:

1. The delay sequence $T_{i}\left(i=1,2,\cdots ,n\right)$ needs to be within U(0,1), to be modulated into a delay sequence satisfying F(x) by reversible transformation function tran(x). In fact, the delay sequence $T_{i}\left(i=1,2,\cdots ,n\right)$ comes from the set $E=\{T:T_{\min}+n\cdot \Delta ,n=0,1,\cdots ,K\}$. $T_{\min}$ represents the minimum interval delay among the packet interval arrival delays. Δ represents the step length between the arrival delays of adjacent packets, which is not uniformly distributed on (0,1). When the sender and receiver synchronize the code table through the controller, $T_{\min}$ and Δ are determined. The delay sequence $T_{i}\left(i=1,2,\cdots ,n\right)$ is represented by vector $\left(j_{1},j_{2},\cdots ,j_{n}\right)j_{i}\in \left[0,1\right]$.
2. A random vector $\left(r_{1},r_{2},\cdots ,r_{n}\right)$ is generated by pseudo random code generator *RAND(x)*. And vector $\left(j_{1},j_{2},\cdots ,j_{n}\right)$ is converted into random vector $\left(j_{1}^{\prime },j_{2}^{\prime },\cdots ,j_{n}^{\prime }\right)$ uniformly distributed over (0,1) by Equation (8).
  (8) $$\begin{array}{c} j_{i}^{\prime }=\left(j_{i}+r_{i}\right)\;\mathrm{mod}\;1 \end{array}$$
3. Finding out the probability density function $f\left(x\right)=F^{\prime }\left(x\right)$ of the distribution function F(x), its domain is (a,b). The domain is divided into *n* parts: $\left(a=a_{0}<a_{1}<a_{2}<\cdots <a_{n}=b\right)$, and the probability *p* of each interval is shown in Equation (9).
  (9) $$\begin{array}{c} p_{i}=\int_{a_{i-1}}^{a_{i}}f(x)dx \end{array}$$
4. Similarly, the interval (0,1) is divided into *n* parts: $\left(0=m_{0}<m_{1}<\cdots <m_{n}=1\right)p_{i}=m_{i}-m_{i-1}$. The vector $\left(s_{1},s_{2},\cdots ,s_{n}\right)$ is used as new delay sequence of packets after calculating by Equation (10).
  (10) $$\begin{array}{c} s_{i}=\frac{\left(a_{i}-a_{i-1}\right)\left(j_{i}^{\prime }-m_{i-1}\right)}{m_{i}-m_{i-1}}+a_{i-1},j_{i}^{\prime }\in \left(m_{i-1},m_{i}\right] \end{array}$$

The watermark is modulated by distribution simulation so that the delay sequence of packets conforms to the normal distribution.

#### 4.2.2. Mapping Synchronization

The coding mapping table needs to change constantly. Only when both sides keep the same mapping can the system run normally. In SACT, we use strict clock synchronization based on NTP protocol to solve the synchronization problem of both controllers. The sending and receiving of NTP timestamp is implemented by UDP.

**Figure 7:**
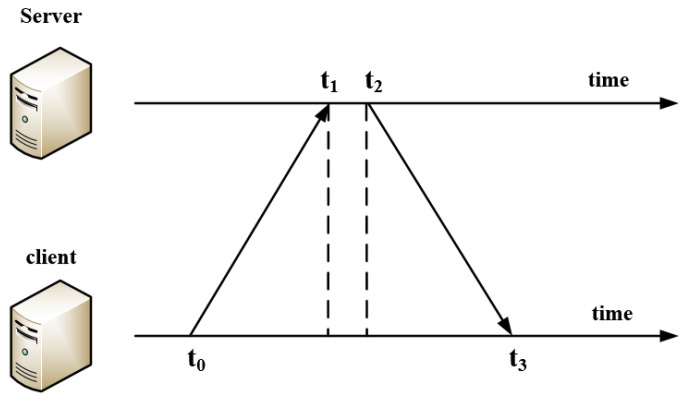
Network Time Protocol (NTP) Synchronization.

As shown in Figure 7, the client calculates its time offset and round-trip delay with the server to synchronize the clock. where $t_{0}$ is the client timestamp when sending the request message, $t_{1}$ is the server timestamp when receiving the request message, $t_{2}$ is the server timestamp when sending the response message, $t_{3}$ is the client timestamp when receiving the response message. Then the round trip delay δ and time offset θ between client and server are shown in Equations (11) and (12).

(11) $$\begin{array}{c} \delta =\left(t_{3}-t_{0}\right)-\left(t_{2}-t_{1}\right) \end{array}$$

(12) $$\begin{array}{c} \theta =\frac{\left(t_{1}-t_{0}\right)+\left(t_{2}-t_{3}\right)}{2} \end{array}$$

The basic process of the module is as follows:

1. SDN controller sends request message to multiple NTP servers.
2. After receiving the response message, the values of δ and θ are statistically analyzed through the filter.
3. Removing the outliers and calculating the estimated time offset from the remaining three best candidate values.
4. Setting the system time and provide timestamp for confirming mapping scheme to complete scheme synchronization.

### 4.3. Watermark Extraction Module

When the data stream arrives at the receiver, SACT extracts and decodes the watermark information through the watermark extraction and decoding module to confirm the communication relationship. After receiving the data stream, the switch passes the information to the SDN controller on the secure channel through the flow table and cache processing mechanism in SDN. Then, the sequence of arrival delay is converted to watermark information by anti jamming mechanism and Viterbi decoding.

The workflow of watermark decoding module is as follows:

1. If the switch receives the TCP/UDP protocol packet, it uploads the key information of the packet to the controller for further processing through *Packet-In* message. If the switch receives other protocol packets, it forwards them directly according to the flow table.
2. After receiving the message, the SDN controller records the arrival time of the next series of homologous packets and calculates the delay sequence of interval arrival $M^{\prime }$.
3. After antijamming processing, the delay sequence $M^{\prime }$ is converted into binary sequence $E^{\prime }$ according to encoding mapping scheme. Then it is converted into binary sequence $B^{\prime }$ according to Viterbi decoding.
4. Calculating the similarity between the sequence code $B^{\prime }$ and the original embedded watermark code *B*.

If the calculated result is within the confidence threshold, the watermark has been embedded in the detected packet stream. That is, there is an association relationship between the two parties of the monitored communication, otherwise there is no communication relationship.

#### 4.3.1. Anti-Interference Mechanism

In the complex network environment, there are network jitters and other factors that affect the arrival delay of data packets, resulting in deviation from the original set delay. In order to solve this problem, a watermark anti jamming mechanism is set up in the system.

The time delay set of interval arrival *E* and the step length Δ between the arrival delays of adjacent packets in the *L-Bits to N-Packets* coding scheme are chosen to modify the watermark with Equation (13).

(13) $$\begin{array}{c} C\left(x\right)=\left\{\begin{array}{c} T_{1},\\ T_{2}+\Delta ,\\ \vdots \\ T_{n}+\Delta \left(n-1\right), \end{array}\right.\begin{array}{c} x\in [T_{1}-\Delta /2,T_{1}+\Delta /2)\\ x\in [T_{2}-\Delta /2,T_{2}+\Delta /2)\\ x\in [T_{n}-\Delta /2,T_{n}+\Delta /2) \end{array} \end{array}$$

In Equation (13), $T_{i}=T_{\min}+\left(i-1\right)\Delta \left(i=1,2,\cdots ,n\right)$. If the interval arrival delay extracted by the controller is *x* and $x\in [T_{i}-\Delta /2,T_{i}+\Delta /2)$, the interval arrival delay embedded in the sender is considered to be $T_{i}$. Thus, the received delay sequence can be processed to obtain the original set sequence.

#### 4.3.2. Viterbi Decoding

SACT system uses convolutional code with constraint length of 9, so there are eight shift registers in the encoder and the status of each register is (0,1). An input sequence is entered at a time, the state of the register affects the others, and the cumulative state is the encoder state. Therefore, there are 28 states in the encoder.

According to the (2, 1, 9) convolutional coding scheme selected by the system, the Viterbi decoder makes 256 cumulative comparison and selection for the received data at a time. Each of the 256 states is followed by two branches (the state of the register is 0 or 1).

Then, they are transferred to two different registers along the two branches to create two paths. The corresponding output of the two paths is compared with the received convolution output, and the one with the smallest Hamming distance is selected to save. This path is called survival path.

Add this survival path to the Hamming distance of the survival path preserved at the previous time, the path selection elimination process is performed for all 256 states. At the end of decoding, the shortest Hamming distance from the 256 surviving paths generated by the above process is selected, and the final decoding output is obtained by backtracking. The algorithm is implemented as follows:

- Suppose that the binary convolution sequence received at time *t* is *s*, the leading state of the first state is p(i,j), and the codeword of the corresponding time is c(i,j),j=1,2.
- Select the branch path, sum it and the measurement of the previous time path:
  (14) $$\begin{array}{c} BM(i,j)=M((c(i,j,1)+1,s(t,1)+1)+M((c(i,j,2),s(t,2)+1) \end{array}$$
  (15) $$\begin{array}{c} PM(i)=\max(PM\_l(p(i,j))+BM(i,j)) \end{array}$$
  Eliminate cumulative overflow:
  (16) $$\begin{array}{c} PM(i)=PM(i)-BM(1,\max\_j(1)) \end{array}$$
- Update current surviving path.
  Read the state data of the last time:
  (17) $$\begin{array}{c} save(i,2:50,:)=save\_l(p(i,\max\_j(i)),1:49,:) \end{array}$$
  Save candidate codewords:
  (18) $$\begin{array}{c} save(i,1,:)=c(i,\max\_j(i),:) \end{array}$$
- Output the decoding result at the current time.
  By calculating the maximum path measure, the maximum likelihood path is obtained:
  (19) $$\begin{array}{c} \max(PM(i)) \end{array}$$
  Output the final path and cycle the codeword of next time:
  (20) $$\begin{array}{c} out(save\_l(\max\_i,50,:)) \end{array}$$

#### 4.3.3. Similarity Judgment

Hamming distance is adopted as the standard for convolutional code decoding. Calculating the cosine similarity of watermark sequences *A* and *B* as the basis of comparison. -

(21) $$\begin{array}{c} sim=\cos\left(\theta \right)=\frac{A\cdot B}{\left||A||*||B|\right|}=\frac{\sum_{i=1}^{n}A\times B}{\sqrt{\sum_{i=1}^{n}{A_{i}}^{2}}\times \sqrt{\sum_{i=1}^{n}{B_{i}}^{2}}} \end{array}$$

Since the range of independent variable parameters $A_{i}$ and $B_{i}$ in Equation (21) is x∈{0,1}, according to the function curve of cosine function, the range of sim value is [0,1]. Comparing the cosine similarity with the set confidence interval threshold parameters. If the result of similarity computation is within the set confidence interval, it is determined that the watermark information embedded in the current detected data stream is related to the source data stream, i.e., there is a communication relationship between the two sides of the current detected communication, otherwise there is no communication behavior.

## 5. Experimental Results

In this section, we evaluate the performance of our system SACT. We deploy SACT system on a server (Ubuntu 16.04 OS, 2.2 GHz, 16 GB RAM, Qingdao, China), using eclipse development tools and Java programming language. We use Floodlight 1.9 (Qingdao, China), Openflow 1.3 (Qingdao, China), Mininet 2.3 (Qingdao, China) and OpenvSwitch 2.8 (Qingdao, China) to build simulation environment.Sniffer is used to capture packets at the receiving end.The corresponding performance tests are carried out in two different test environments: LAN and WAN. Three aspects are tested and analyzed: detection rate analysis, false alarm rate analysis, and antiattack analysis.

### 5.1. Detection Rate

The interval arrival delay of packets is a random variable with normal distribution in the process of network transmission. Different network congestion leads to different normal distribution (different mean and standard deviation) of interval arrival delay. In the experiment, in order to simulate the influence of network jitter under different normal distribution on the detection rate of the system, the network jitter time satisfying different normal distribution is added to the packet delay sequence.

**Figure 8:**
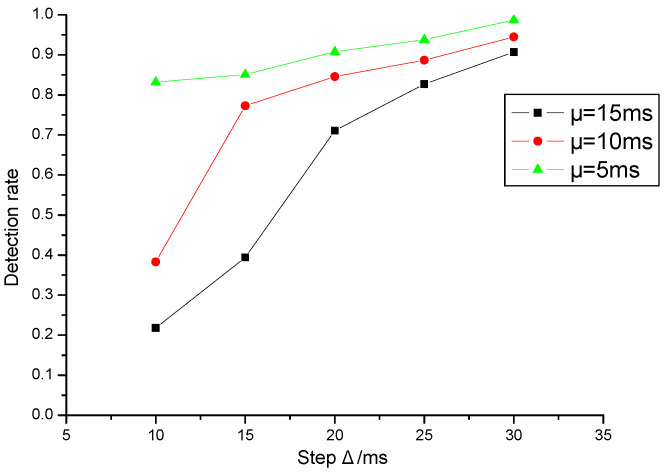
Effect of network jitter on detection rate.

Setting the standard deviation (σ=4), respectively test the detection rate of different watermark interval delay step Δ when the average jitter is 5 ms, 10 ms and 15 ms. A total of 8000 bits of information are transmitted. Setting the threshold parameter of confidence interval when determining cosine similarity as [0.8,1], then the result is shown in Figure 8.

**Figure 9:**
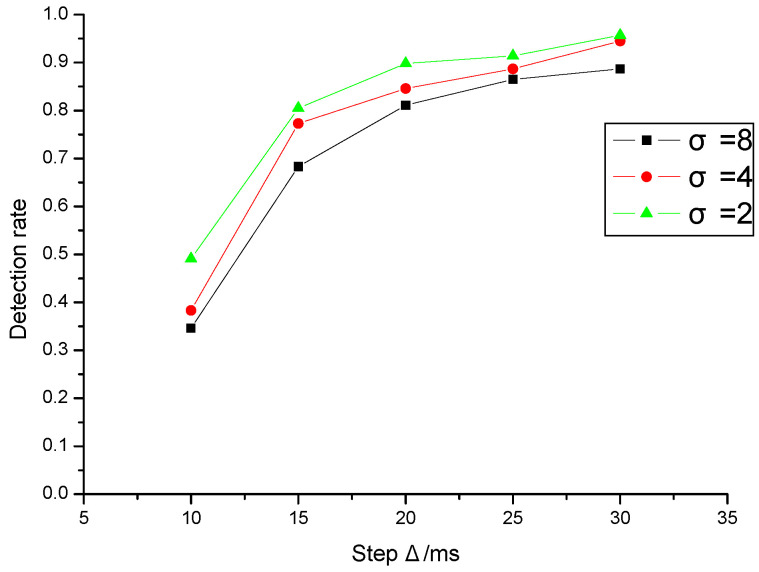
Effect of standard deviation of network jitter on detection rate.

Figure 9 is the effect of different standard deviations (σ=2,4,8) on the watermark detection rate when the average network jitter is given (μ=10 ms). The experimental results show that the smaller the standard deviation of network jitter is, the less the influence on watermark detection is, and the higher the detection rate is. The experimental results can further prove that the larger the watermark interval delay step is, the smaller the impact of network jitter on watermark detection.

**Figure 10:**
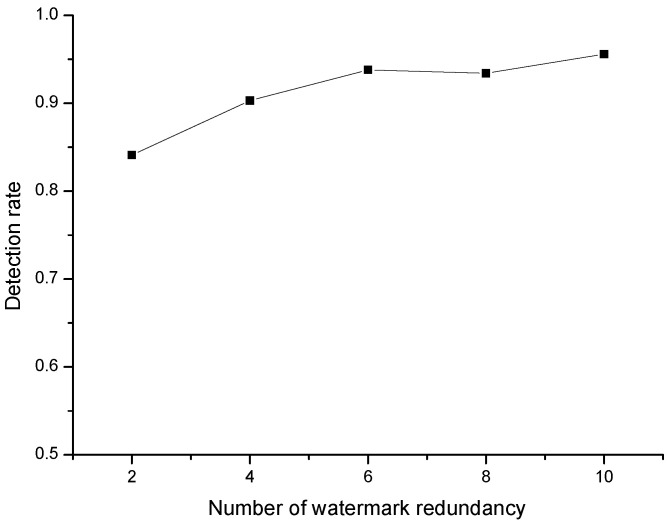
Effect of watermarking redundancy times.

Watermark redundancy refers to the situation that the same watermark information is embedded into the carrier data stream several times in succession. Usually, both sides of anonymous communication keep continuous communication for a certain period of time. The watermark information comes from the port identification information of switch, so the redundancy of watermark information will be produced. Therefore, it is necessary to test the influence of watermark redundancy on detection rate. When the network jitter is fixed (σ=4,μ=10), the influence of watermark redundancy times *r* on detection rate is tested. The results are shown in Figure 10. The results show that the more redundant the watermark is, the higher the detection rate is. When the number of redundant watermark is more than 5, the detection rate is higher than 0.9. In conclusion, the anonymous communication tracking model proposed in this paper can work reliably and stably in our simulated networks.

### 5.2. False Alarm Rate

**Figure 11:**
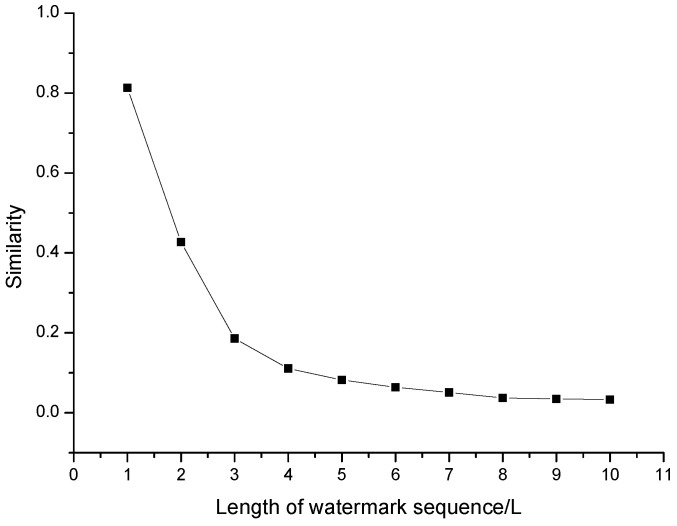
Effect of watermarking redundancy times.

False alarm rate is also an important standard to measure watermark tracking scheme. If the controller attempts to extract the watermark information from the data stream without any watermark information, it is possible to cause some false positives. For the data stream without watermark information, the controller extracts the watermark and judges the similarity with the expected watermark information. If it matches the threshold parameter of confidence interval, it is considered that the watermark information is embedded in the detected data stream and leads to misjudgment. Otherwise, it is determined that the current detected data stream is not embedded with watermark information. The experiment of false alarm of our system is shown in Figure 11.

In this experiment, we send the watermark sequence with step Δ=20 ms between the arrival delay of adjacent packets, and the minimum interval arrival delay is $T_{\min}=30$ ms. We set the confidence interval threshold parameter of cosine similarity determination to [0.8,1]. At the same time, we simulate the network jitter distribution with mean value of μ=10 and standard deviation of σ=2. The experimental results show that if the length of embedded watermark sequence is 1 L (unit length), we detect and determine the watermark when the controller does not embed any watermark, the average similarity is close to the lower boundary of the confidence interval threshold, which leads to a high error rate.

With the increase of the length of the watermark sequence, the detection similarity of the watermark sequence decreases rapidly. When the length of the watermark sequence is larger than 2 L, the detection similarity is much lower than the lower boundary of the confidence interval threshold, and the false alarm rate is low. Therefore, SACT has a low false positive rate and can be used in anonymous communication tracking.

### 5.3. Anti Attack Ability

Our system relies on distributed simulation mechanism and mapping synchronization mechanism to ensure our anti attack ability. Therefore, we need to carry out experimental test and analysis from these two aspects.

#### 5.3.1. Distributed Simulation Test

**Figure 12:**
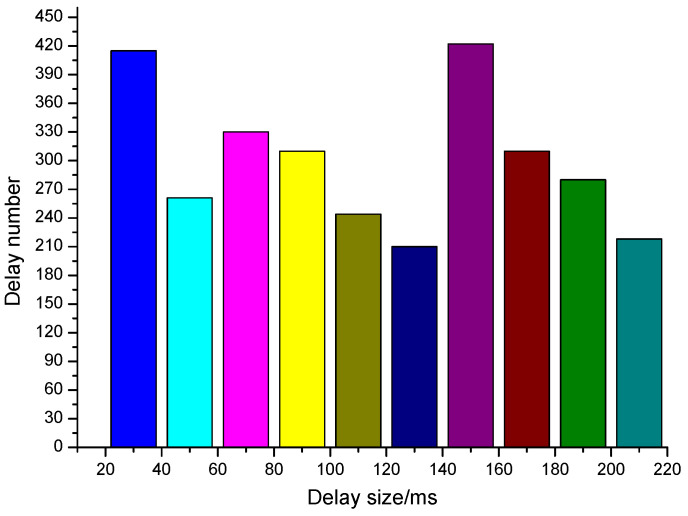
Time delay distribution before simulation of distribution.

In the experiment, the normal distribution of data flow is (σ=4,μ=20). We compare the SACT system with the Time Lord model proposed in Castillo et al. [17]. In the experiment, 3000 interval arrival delays are sent through the Time Lord model. The step size between the arrival delays of adjacent packet is Δ=20 ms, and the minimum interval arrival delay is $T_{\min}=30$ ms. The interval arrival delay distribution of carrier data stream is shown in Figure 12. It can be found that its distribution law is completely different from normal data flow, and it is easy to be attacked.

SACT sends the same number of interval arrival delay at the sender, and the step size and minimum interval arrival delay between adjacent packets are consistent with Time Lord model. It is transformed into the distribution state (σ=4,μ=20) of normal data stream and embedded into data stream for transmission. After receiving the data stream, the receiver controller extracts the delay sequence and decodes it to obtain the watermark information.

**Figure 13:**
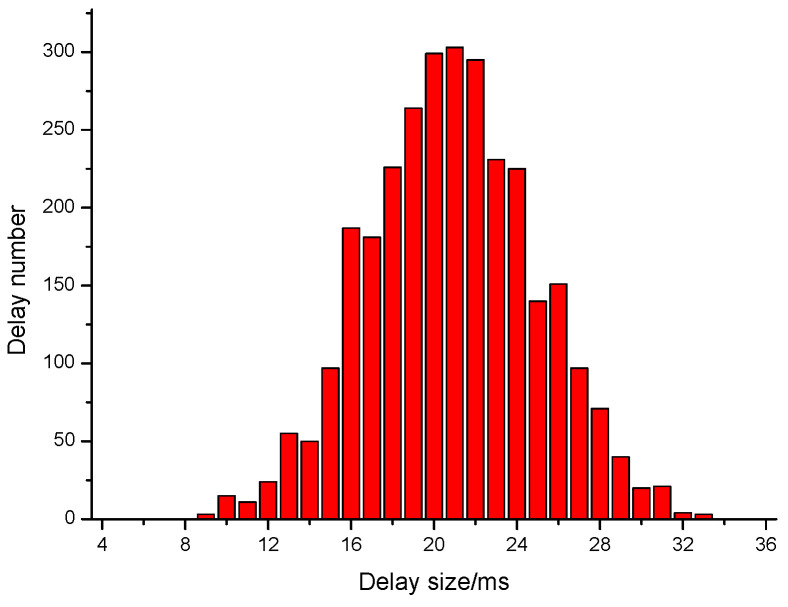
Time delay distribution after simulation of distribution.

After simulating and modulating the delay sequence of interval arrival through the distributed simulation mechanism, the delay distribution is shown in Figure 13. It can be seen that it basically matches the distribution state of delay sequence of normal data stream. SACT improves the anti detection ability of watermark information and realizes the covert tracking of anonymous communication.

#### 5.3.2. Mapping Synchronization Test

In the experiment, we get the data stream in the network by intercepting attack. When mapping dynamic synchronization is not added, the mapping relationship in the controller is static. For example, the MAC address of one side of network communication is C8−D3−FA−E2−F2−88, the minimum interval arrival delay of the current network is $T_{\min}$ = 30 ms, and the interval sequence step size is Δ=20 ms. The mapping relationship after watermark transformation is shown in Table 2.

**Figure 14:**
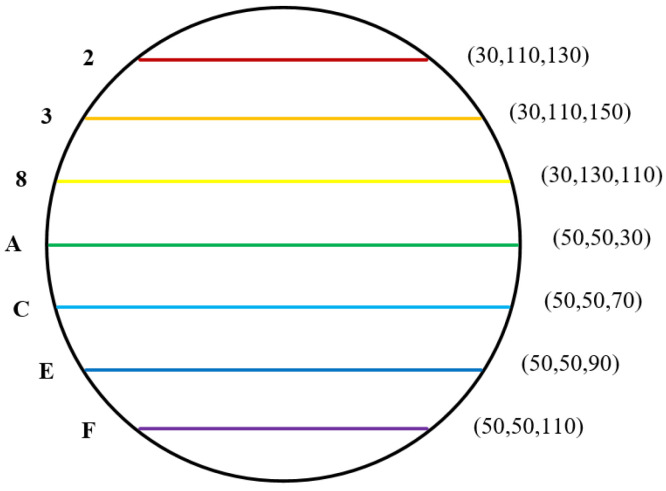
Mapping graph without dynamic transition synchronization.

In order to analyze the problem more intuitively, we give the mapping diagram, as shown in Figure 14. The mapping relationship is a stable one-to-one relationship in the whole monitoring process. When the attacker intercepts the system, it is relatively easy to get the potential watermark conversion relationship. It is easier for the attacker to find the watermark and even get the watermark content, which makes the whole system in an insecure state.

**Figure 15:**
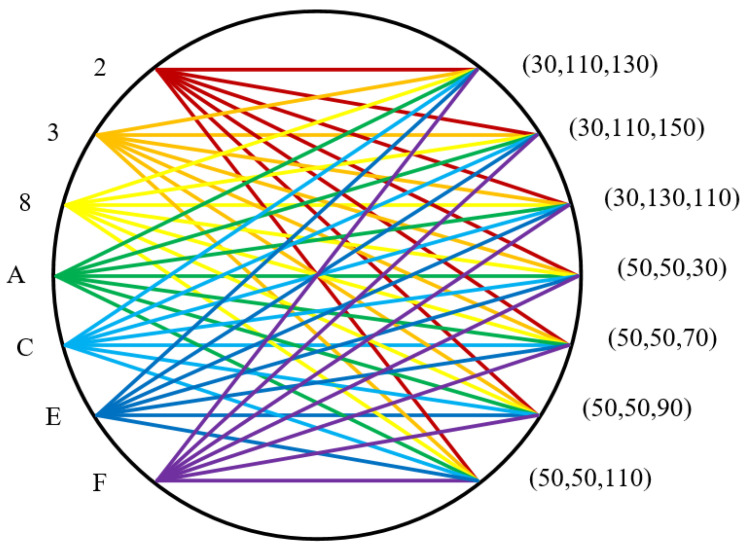
Mapping graph with dynamic transition synchronization.

We add mapping dynamic synchronization, changing the current mapping scheme by using the timestamp returned by NTP server. Thus, the diversity of communication traffic delay sequence is realized, which makes it difficult for the attacker to analyze and obtain the information beneficial to his attack, so as to confuse the attacker. The same network environment is used in the experiment. As the monitoring time goes on, the mapping relationship will become more and more complex. The mapping diagram at this time is shown in Figure 15.

In the field of anonymous communication, whether anonymous communication can be realized safely and effectively is the main evaluation criterion. In addition, the evaluation criterion include cost, efficiency, and deployment difficulty. We made a horizontal comparison between our mechanism and the work in Castillo et al. [17] and Xu et al. [19], and the result is shown in the Table 3.

In Table 3, we can see that our system SACT is dynamic and easy to deploy on the basis of maintaining effective anonymous communication, which ensures our system has higher security and flexibility.

Summary: We introduce the system test environment, then test it from three aspects: detection rate, false alarm rate, and antidetection ability. The experimental results show that the proposed scheme can maintain a good detection rate in most of the our conditions, making the detection similarity of the watermarked data stream far lower than the lower boundary of the confidence interval threshold, and the false alarm rate is low. It can accord the distribution of the watermark delay sequence with the normal data flow. In addition, we make the mapping relationship disordered, thus improving the ability of antidetection and hidden tracking of watermark information.

## 6. Discussion

Through the experimental results, we can know that in the experimental environment we set up, the SACT system operates stably and efficiently as we expected. In methodology, we took into account the actual environment and made corresponding analysis of theoretical. Our experimental environment also simulates the real environment as much as possible, including network jitter, delay distribution and so on. However, there are still some aspects that we need to think about and discuss:

1. NTP synchronization is simple and efficient with mature processing method clock skew. However, the security issues of NTP servers have not been fully resolved. We are considering finding more secure and efficient synchronization methods in the future, or making improvements to NTP servers, such as adding a blockchain mechanism.
2. Algorithms of convolutional encoding and decoding can be further optimized. Furthermore, we will promote the system in the future.

## 7. Conclusions

Anonymous communication technology plays an important role in protecting information security. In traditional networks, after the deployment of an anonymous communication system, if the configuration of firewalls, routers, and switches in the network needs to be modified, it will be a complicated, costly, and time-consuming task. The traditional tracking technology of network watermark relies on the deployment of tracking equipment, and additional equipment such as a watermark embedder must be added to the corresponding network environment to take over all network traffic. It has caused additional resource consumption and easily aroused the vigilance of criminals, which has prevented large-scale implementation.

Targeting the difficulty to deploy poor reusability and low scalability of the traditional network, in this paper, we studied the Software Defined Network and network watermarking. By taking advantage of SDN to separate the control layer and the data layer, we integrated the network watermark with the SDN to achieve the tracking of covert communication. Hiding relevant details from users, SDN can switch the current state at any time without changing any deployment. Making use of its good expansibility, this paper proposes a watermarking scheme to further improve the detection and anti detection of the tracking watermark, which lays a good foundation for the application and expansion of the watermark. The security and effectiveness of the SACT are verified through detection rate, false alarm rate, and anti attack test, which proves that the system can solve the deployment problem of anonymous tracking and improve the availability and scalability of covert communication.

## Figures and Tables

**Table 1:** L-Bits to N-Packets Codes.

m	T/ms
10	60
01	80
11	100
00	120

**Table 2:** Coding mapping relation.

Character	Binary Code	Delay Sequence
2	0011 0010	(30,110,130)
3	0011 0011	(30,110,150)
3	0011 1000	(30,130,110)
A	0100 0001	(50,50,30)
C	0100 0011	(50,50,70)
E	0100 0101	(50,50,90)
F	0100 0110	(50,50,110)

**Table 3:** Comparison of features of different anonymous communication channels.

Feature	CTC	Non-Zero	SACT
effective	Yes	Yes	Yes
concealment	Yes	Yes	Yes
low-cost	No	Yes	Yes
dynamic	No	No	Yes
Easy to deploy	No	No	Yes
